# Neurohumoral mechanisms associated with orthostasis: reaffirmation of the significant contribution of the heart rate response

**DOI:** 10.3389/fphys.2014.00236

**Published:** 2014-06-30

**Authors:** Victor A. Convertino

**Affiliations:** U.S. Army Institute of Surgical Research, Research DivisionJBSA Fort Sam Houston, TX, USA

**Keywords:** orthostatic tolerance, blood pressure regulation, lower body negative pressure, parasympathetic activity, sympathetic activity, propranolol, atropine

## Abstract

The inability to compensate for acute central hypovolemia underlies the clinical development of orthostatic hypotension and instability (e.g., syncope). Although neuro-humoral control of both cardiac output and peripheral vascular resistance contributes to hemodynamic stability during orthostasis, a notion has been proposed that the failure of adequate peripheral vascular constriction rather than cardiac responses represents the primary mechanism underlying the development of orthostatic intolerance. This review article provides an opportunity to present compelling evidence captured over the past 30 years in our laboratory to support the concept that neural-mediated tachycardia during orthostasis in healthy individuals represents a critical response to tolerating acute reduction in central blood volume in addition to, and independent of, peripheral vascular constriction. In this review paper, data are presented from experiments using graded lower body negative pressure (LBNP) as a method to induce orthostatic intolerance in two experimental human models: (1) comparison of heart rate and autonomic responses in individuals with relatively high and low tolerance to LBNP; and (2) vagal and sympathetic blockade of cardiac neural control. These experiments revealed that: (1) greater elevations in heart rate are associated with higher orthostatic (LBNP) tolerance; (2) higher orthostatic heart rate is associated with greater sympathetic nerve activity and withdrawal of vagally-mediated cardiac baroreflex response; and (3) non-specific sympathetic blockade causes a pronounced reduction in heart rate and LBNP tolerance. Cardiac parasympathetic withdrawal contributes to protection against development of hypotension during the initial seconds of transition to an orthostatic challenge, while the primary mechanism by which tachycardia defends orthostatic stability in healthy subjects for extended durations is mediated predominantly through sympathetic adrenergic control.

## Introduction

Clinical orthostatic hypotension manifested by intolerance to acute reductions in central blood volume can result in physical debilitation of patients who have been confined to bed or with autonomic dysfunctions (Robertson and Biaggioni, 1995). Perhaps of equal interest is the predisposition to orthostatic intolerance in apparently healthy individuals (Sather et al., 1986; Convertino, 1987; Convertino and Sather, 2000b). The inability to compensate for acute central hypovolemia (e.g., action of moving quickly from lying in bed to standing) underlies the rapid onset of hypotension and a potentially inadequate cerebral perfusion. Although multifactorial and complexly integrated neuro-humoral control of both cardiac output and peripheral vascular resistance ultimately contributes to hemodynamic stability during orthostasis, a preponderance of evidence has focused on the failure of adequate peripheral vascular constriction as the primary mechanism underlying orthostatic intolerance in healthy individuals under conditions of low cardiac filling. For instance, the blockade of a heart rate response failed to affect mean arterial pressure during head-up tilt compared to a non-drug condition (Tyden, 1977). This observation was used to advance the notion that peripheral vascular constriction rather than cardiac responses represents the primary mechanism essential to maintaining arterial pressure during orthostatic challenge (Rowell, 1993). This notion was reinforced by the observation that blunted elevations in peripheral vascular resistance with similar tachycardia distinguished astronauts who could not complete a stand test following spaceflight from those who completed the test without symptoms (Buckey et al., 1996).

Although elevated heart rate is a typical reflex response to orthostasis, the importance of tachycardia as a compensation for reduced stroke volume may be less clear. There are numerous published data that provide various hemodynamic comparisons of individuals who display orthostatic intolerance (i.e., syncope) during clinical stand or tilt tests with those who do not faint (Rowell, 1993; Robertson and Biaggioni, 1995; Buckey et al., 1996; Cooper and Hainsworth, 2001, 2002; Fu et al., 2012). However, differences in mechanisms of maximal compensation cannot be completely defined using tests that fail to elicit “orthostatic” tolerance in all subjects (e.g., stand or tilt). As a somewhat unique approach, our laboratory has focused on the use of lower body negative pressure (LBNP) so that comparisons of physiology in individuals with high and low tolerance to central hypovolemia could be studied at their maximal level. As such, it is the objective of this review article to present evidence obtained during various experiments conducted in our laboratory on healthy humans over the past 30 years in an attempt to demonstrate the significance of autonomically-mediated tachycardia during orthostasis. Specifically, the data support the notion that heart rate represents a physiologically important response to tolerating acute reduction in central blood volume in addition to and independent of peripheral vascular constriction.

## Experimental model of orthostasis

In this review paper, data are presented from experiments using graded LBNP as a method to induce “orthostatic” intolerance. The primary hemodynamic response to a “standard” orthostatic challenge such as standing or head-up tilt is a translocation of blood from the central blood vessels and heart to the large veins in the lower extremities caused by the effect of gravity, with the magnitude of volume shift being an important determinant of the physiological response (Convertino, 1987, 2001a). In this regard, there is compelling evidence that the cardiovascular responses to LBNP, standing and head-up tilt are both qualitatively and quantitatively similar (Blomqvist and Stone, 1983), particularly for peripheral vascular resistance and heart rate (Gilbert and Stevens, 1966; Convertino, 1993). To study the neurohumoral reflex mechanisms associated with the response to orthostasis, the use of experimental techniques that cause reproducible and quantifiable volume redistribution similar to standing is necessary. Perhaps more important, a major limitation to the investigations of contributing mechanisms to hemodynamic competence using stand or tilt tests is the inability to define orthostatic tolerance of the cardiovascular system in all individuals. This limitation can result in incorrect or misleading interpretations (Tyden, 1977; Rowell, 1993; Buckey et al., 1996; Convertino and Cooke, 2005). LBNP has proved to be a very effective technique for the study of cardiovascular reflex mechanisms associated with orthostatic intolerance (Convertino, 2001a). Among several distinct and unique design advantages, LBNP produces a gravity-independent redistribution of venous volume with minimal muscular activity or vestibular stimulation while the subject remains in the supine posture. As such, the data presented in this review were generated from experiments in which LBNP was used to elicit pre-syncopal episodes in all individuals so that the contribution of neutrally controlled tachycardia to orthostasis could be more accurately assessed.

## High vs. low tolerant individuals

One prominent observation evident from our laboratory experiments is the existence of a subset of individuals who have relatively low tolerance to reduced central blood volume. In clinical or operational settings, these individuals have been referred to as non-finishers (Buckey et al., 1996), fainters (Convertino, 1993), or low tolerant (Sather et al., 1986; Convertino and Sather, 2000b; Rickards et al., 2011; Convertino et al., 2012). Historically, individuals have been classified as having low (fainters) or high (non-fainters) orthostatic tolerance based on development of syncope during standing (Convertino, 1993). The results from previous experiments indicate that similar heart rate responses to standing mirror those induced by 50 mmHg LBNP (Convertino, 1993). Based on previous investigations in our laboratory (Sather et al., 1986; Convertino and Sather, 2000b; Rickards et al., 2011; Convertino et al., 2012), subjects who participated in our experiments were categorized as having high orthostatic tolerance based on entrance into the -70 mmHg LBNP level while individuals who failed to complete -60 mmHg of LBNP were categorized as low tolerant.

The comparison of individuals with high and low tolerance to acute central hypovolemia can be used to identify neurohumoral contributions to orthostasis. In this review, results generated from our laboratory LBNP experiments designed to determine tolerance in all subjects will be used to describe the role(s) of autonomic control of heart rate responses to maintain adequate perfusion during acute progressive reductions in central blood volume.

## Contribution of peripheral vasoconstriction during orthostasis

**Figure 1:**
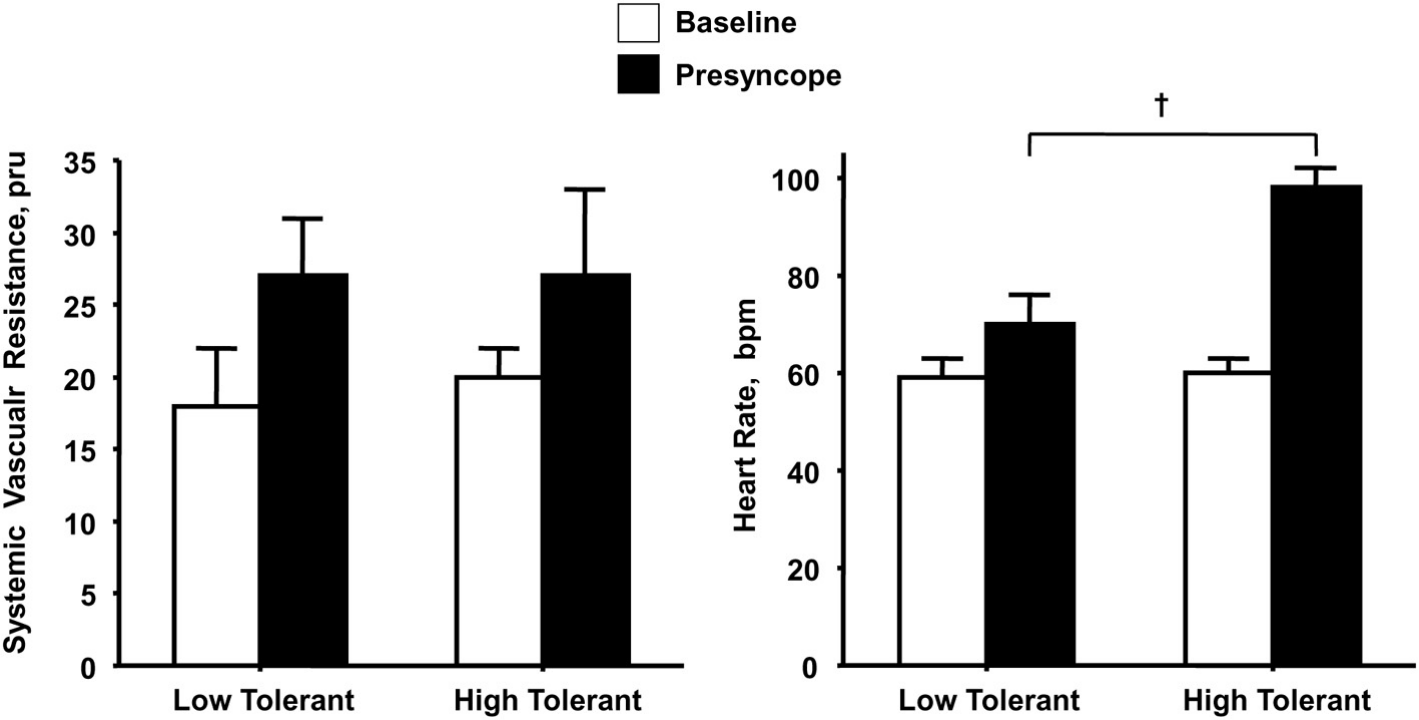
**Systemic vascular resistance and heart rate responses at baseline (open bars) and onset of presyncope (closed bars) in low (*N* = 5) and high (*N* = 6) LBNP tolerant individuals**. Symbols are mean ± 1 s.e.m. for each group. Data from Convertino and Sather (2000a).

If peripheral vasoconstriction rather than cardiac responses represents the primary mechanism for maintaining adequate perfusion pressure during orthostatic challenge, one would expect a significantly greater increase in peripheral vascular resistance in subjects with high compared to low LBNP tolerance with little difference in elevated heart rate response. Similar to others (Buckey et al., 1996; Cooper and Hainsworth, 2002), we have consistently reported that increased vascular resistance represents a fundamental compensatory mechanism for maintaining arterial pressure in response to reduced cardiac filling induced by maximal levels of LBNP (Convertino et al., 1986, 2012; Sather et al., 1986; Convertino, 1987, 1993, 1998; Convertino and Sather, 2000b). Consistent with the notion that this peripheral vasoconstriction contributes importantly to maintaining blood pressure and tolerance to orthostasis, we have reported that measures of systemic vascular resistance were greater in high compared to low tolerant subjects (Sather et al., 1986; Convertino and Sather, 2000b; Convertino et al., 2012) coincident with greater elevations in the circulating vasopressor hormones vasopressin, angiotensin, and norepinephrine (Convertino, 1993; Convertino and Sather, 2000b). However, the important difference in our data is that it includes peripheral vascular responses at tolerance in ALL subjects. Since the other studies fail to elicit a response at syncope in all subjects, there is no way to determine that the heart rate response in patients who do not experience syncope would not have been greater. In contrast, the elevation in maximal heart rate during LBNP elicited in our experiments has proven to be consistently higher in high compared to low tolerant individuals (Sather et al., 1986; Convertino, 1993; Convertino and Sather, 2000a,b; Convertino et al., 2012). In fact, in one experiment (Convertino and Sather, 2000a) greater LBNP tolerance in one group of subjects was associated with greater tachycardia but no difference in systemic peripheral resistance compared to a group of low tolerant subjects (Figure 1). Taken together, data from our laboratory suggest that orthostatic tachycardia must also represent an important contributing mechanism to determining the ability to compensate for reduced central blood volume during orthostasis. This notion is further supported by an independent investigation that demonstrated significantly reduced cardiac output driven predominantly by lower heart rate contributed to pre-syncope in some individuals without changes in peripheral vascular resistance (Fu et al., 2012).

## Role of vagally-mediated control of heart rate

We have consistently reported in cross-sectional population comparisons of individuals that compromised ability to maintain blood pressure in orthostatic settings is associated with an attenuated vagally-mediated cardiac baroreflex at baseline rest (Convertino et al., 1991; Convertino, 1998, 2001b). This finding is particularly compelling in support of the importance of the heart rate response to orthostasis when there is no difference in the magnitude of increase in peripheral resistance (Convertino and Sather, 2000a; Convertino, 2001b). The association between attenuated reflex response of heart rate and orthostatic hypotension in cross-sectional population comparisons led to investigations that utilized longitudinal changes in the vagally-mediated cardiac baroreflex of the same individuals.

**Figure 2:**
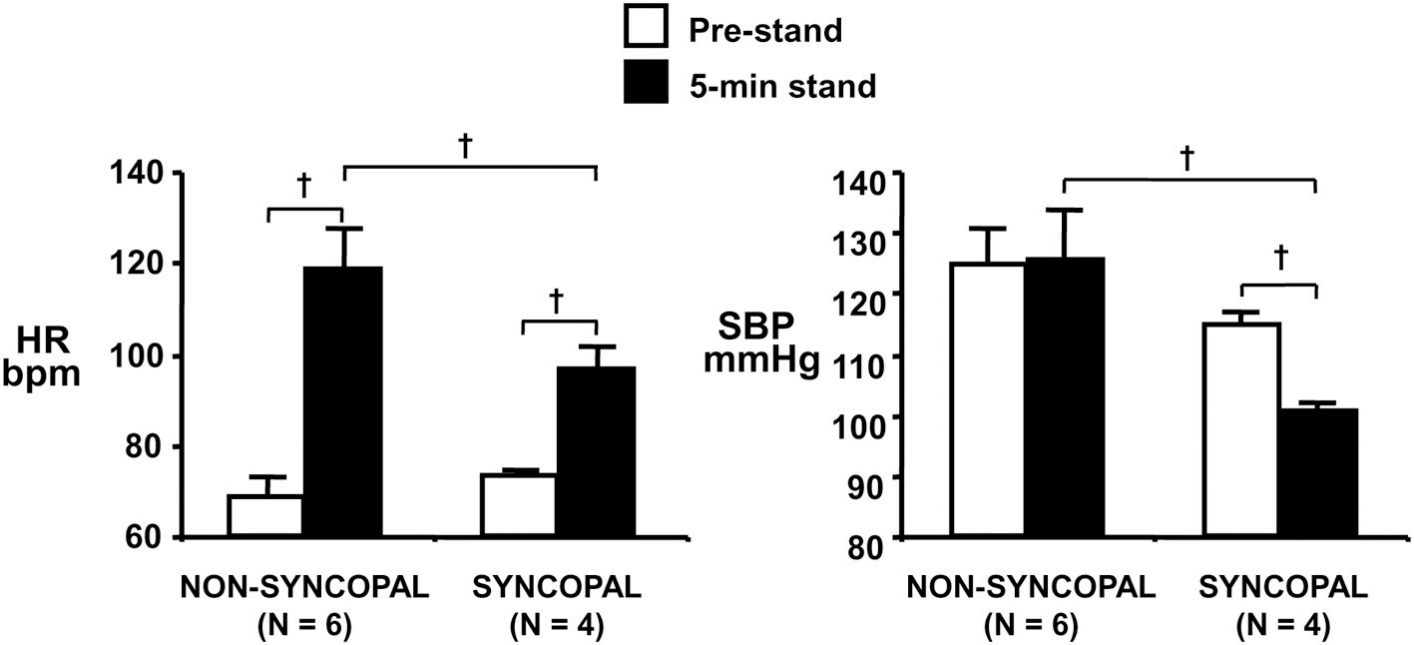
**Heart rate (HR, left panel) and systolic blood pressure (SBP, right panel) during supine pre-stand (open bars) and at 5 min of standing (solid bars) in subjects who did or did not experience syncopal symptoms during a stand test following 30 days of bed rest**. Symbols are mean ± 1 s.e.m. Figure from Convertino et al. (1990). ^†^indicates *P* < 0.05.

**Figure 3:**
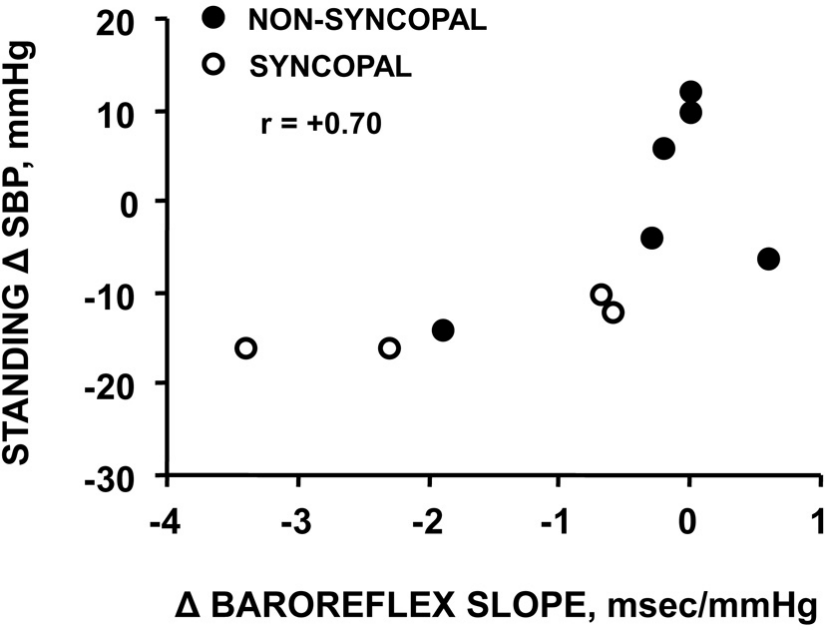
**Relationship between changes in carotid-cardiac baroreflex sensitivity (ΔBaroreflex slope) and standing systolic blood pressure (Standing Δ SBP) in subjects who did or did not experience syncopal symptoms during a stand test following 30 days of bed rest**. Figure from Convertino et al. (1990).

Using confinement to prolonged bed rest as a model to chronically reduce the cardiac baroreflex sensitivity (Convertino et al., 1990), we found that 4 of ten subjects who experienced syncope when they stood immediately at the end of their confinement displayed clinically significant orthostatic hypotension associated with a depressed heart rate response (Figure 2). A lower heart rate in the presence of greater baroreceptor stimulation (i.e., lower arterial blood pressure) suggests an association between a depressed baroreflex response and development of syncope. This association was reinforced by the striking relationship between a greater reduction in baroreflex sensitivity (maximal slope of the stimulus-response reflex relationship between arterial pressure and R-R interval) and a larger hypotension during post-bed rest standing across the 10 individual subjects (Figure 3). In the opposite direction, enhancing cardiac baroreflex sensitivity by intense exercise (Engelke et al., 1994, 1996) or repeated exposure to increased gravity acceleration (Convertino et al., 1998) was associated with more stable blood pressure (i.e., less hypotension) during orthostasis. Although not causal, the relationship between change in baroreflex sensitivity and magnitude of hypotension infers the possibility that vagal control of heart rate has a significant role in the regulation of orthostatic blood pressure.

Our experiments revealed that acute progressive reductions in cardiac baroreflex sensitivity induced by LBNP reflect vagal withdrawal (Cooke and Convertino, 2005), and that the maximum reduction in baroreflex sensitivity from baseline is defined as the physiological “reserve” for vagal withdrawal (Engelke et al., 1996; Convertino et al., 2012). If vagal control of heart rate contributes importantly to blood pressure regulation during an orthostatic challenge, then one might expect a greater reserve to withdraw parasympathetic activity (i.e., greater reduction in cardiac baroreflex sensitivity) in high compared to low tolerant individuals. Indeed, our recent experiments have substantiated such a relationship as evidenced by a higher baseline with greater reduction in spontaneous cardiac baroreflex sensitivity in high compared to low tolerant subjects following presyncopal-limited LBNP (Convertino et al., 2012). These results support the notion that a greater reserve for baroreflex-mediated vagal withdrawal represents a mechanism underlying greater elevations in heart rate in individuals with high orthostatic tolerance.

**Figure 4:**
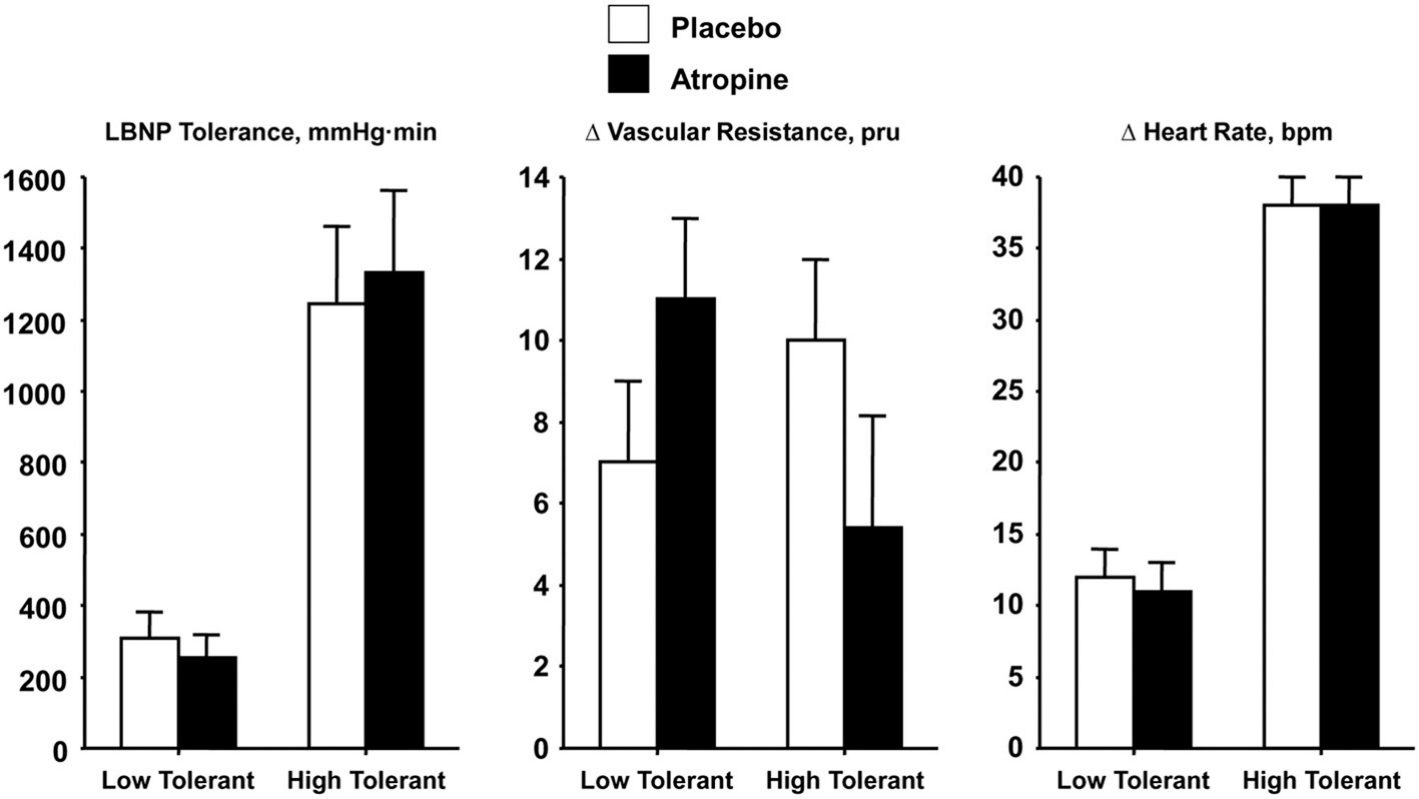
**LBNP tolerances and changes (Δ) in peripheral vascular resistance and heart rate during placebo (open bar) and atropine (closed bar) treatments in low (*N* = 5) and high (*N* = 6) LBNP tolerant individuals**. Bars and lines represent mean ± 1 s.e.m. Data from Convertino and Sather (2000a).

Ultimately, a cause-effect relationship between vagal control of the heart rate response to orthostasis and tolerance must be demonstrated. Such an experiment requires a comparison of hemodynamic responses in orthostatically tolerant and intolerant subjects who have all reached syncopal endpoints while under the influence of cardiac cholinergic blockade. If the magnitude of vagally-controlled tachycardia represents an important mechanism for blood pressure regulation during orthostasis, then blockade of parasympathetic mediated chronotropic responses should elicit syncopal symptoms at lower orthostatic stress in both high and low tolerant subjects. With such an approach, we compared cardiovascular responses in 11 healthy men, categorized as having high (*N* = 6) or low (*N* = 5) tolerance to presyncopal-limited LBNP, before and after atropine administration (Convertino and Sather, 2000a). Consistent with our previous findings, average baseline cardiac baroreflex sensitivity of the high tolerant subjects (−3.8 bpm · mmHg^−1^) was 3-fold greater than that of the low tolerant subjects (−1.2 bpm · mmHg^−1^). Consistent with the notion that baroreflex-mediated tachycardia contributes importantly to hemodynamic stability during orthostatic stress, it is not surprising that atropine failed to reduce LBNP tolerance since the cardiac baroreflex response (i.e.,ΔHR/ΔMAP) was also unaltered (Figure 4, right panel) with similar reductions in MAP (Convertino and Sather, 2000a). These results reinforced the importance of heart rate *response* (i.e., heart rate reserve) rather than absolute heart rate as a compensation mechanism contributing to orthostatic competence. In conditions when heart rate is less than 100 bpm, vagal withdrawal contributes to the elevation of heart rate (Mosqueda-Garcia, 1995). However, in the absence of vagally-mediated cardiac control, the results from our atropine experiments suggest that elevated heart rate is not solely dependent on parasympathetic withdrawal, but that the sympathetic limb of heart rate control represents redundancy to maintain an appropriate reflex tachycardia during orthostasis.

## Role of sympathetically-mediated control of heart rate

**Figure 5:**
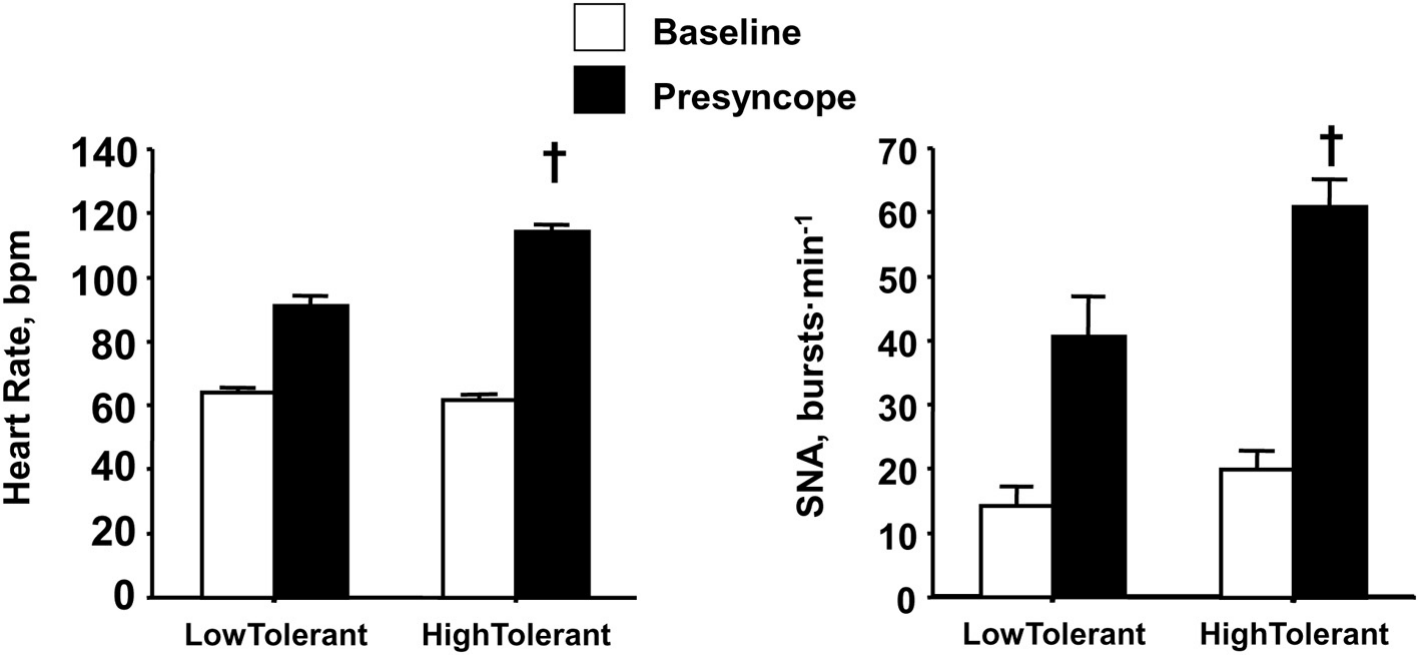
**Heart rate and sympathetic nerve activity (SNA) at baseline (open bars) and pre-syncope (closed bars) in high and low tolerant subject groups**. Bars and lines represent mean ± 1 s.e.m. ^†^Indicates *P* ≤ 0.041 compared with corresponding low tolerant value. Figured modified from Convertino et al. (2012).

Similar to the reserve for vagal withdrawal, the maximum increase in sympathetic nerve activity (SNA) from baseline to orthostatic intolerance reflects the physiological “reserve” for sympathetic activation of the heart. If sympathetic control of heart rate contributes importantly to blood pressure regulation during an orthostatic challenge, than one might hypothesize a greater reserve to increase sympathetic activity in high compared to low tolerant individuals. Consistent with this hypothesis, experiments conducted in our laboratory provided direct measures from peroneal nerves in humans during progressive LBNP to the point of presyncope (Convertino et al., 2012), and demonstrated greater SNA was associated with higher maximal orthostatic heart rates and orthostatic tolerance (Figure 5). Although SNA was obtained from muscle (i.e., MSNA) in these experiments, there is compelling evidence to suggest that MSNA reflects cardiac SNA since multiple firing rates of sympathetic outflow to the skeletal muscle vasculature is associated with high cardiac norepinephrine spillover (Wallin et al., 1992; Lambert et al., 2011). The two-fold greater elevation in SNA from baseline to pre-syncope observed in high tolerant subjects represented a greater SNA reserve when compared to low tolerant subjects. In the absence of a robust SNA and heart rate reserve, low tolerant individuals tolerated a 40% reduction in stroke volume before the onset of presyncope. In contrast, greater reserve for increasing SNA and heart rate enabled high tolerant individuals to maintain cardiac output and blood pressure, and delay the onset of presyncope despite a 56% reduction in stroke volume.

**Figure 6:**
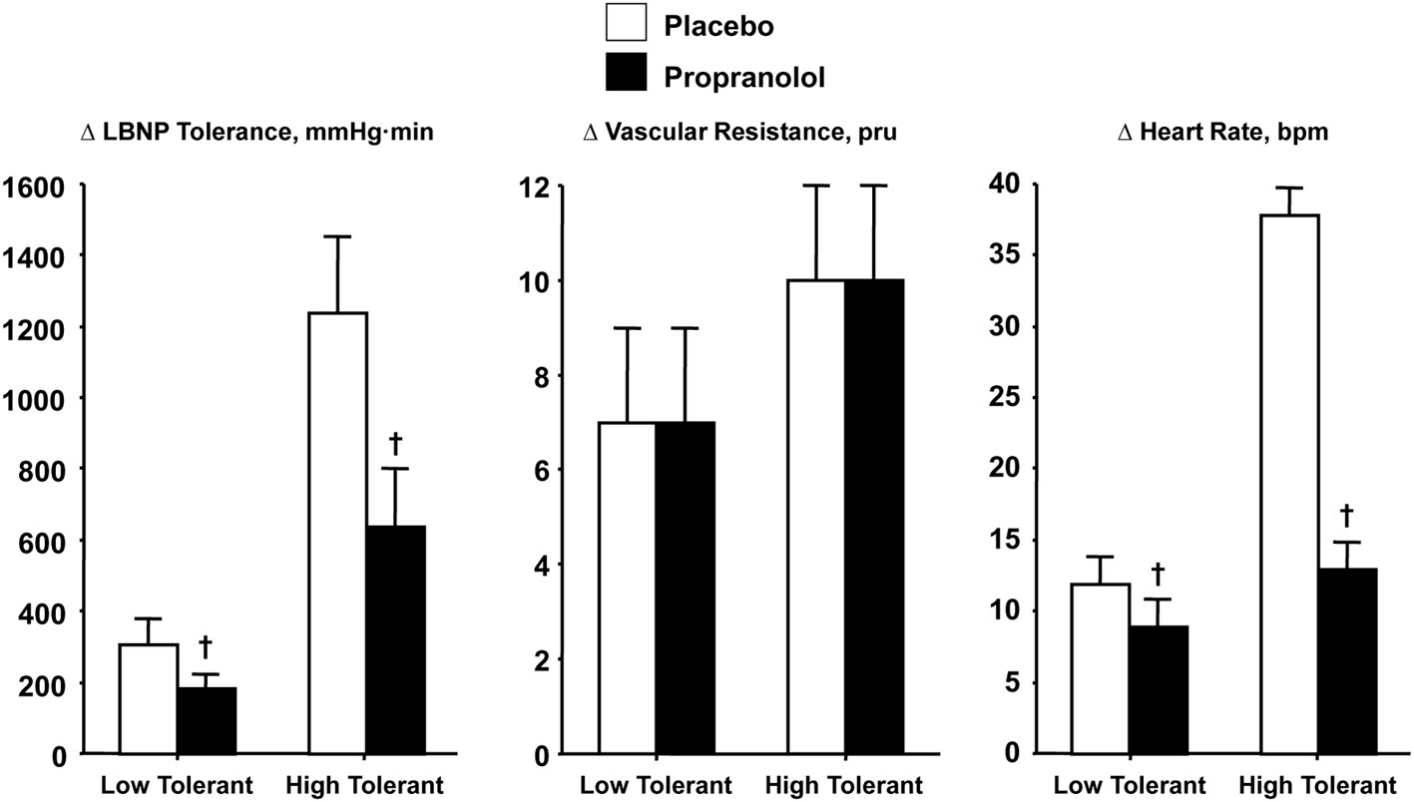
**LBNP tolerances and changes (Δ) in peripheral vascular resistance and heart rate during placebo (open bar) and propranolol (closed bar) treatments in low (*N* = 5) and high (*N* = 6) LBNP tolerant individuals**. Symbols are mean ± 1 s.e.m. ^†^Indicates *P* < 0.05 compared to placebo treatment. Data from Convertino and Sather (2000a).

Verification that increased SNA is causal to elevated heart rate during orthostasis was demonstrated with comparisons of heart rate and LBNP tolerance responses in high and low tolerant subjects who have all reached syncopal endpoints while under the influence of beta-adrenergic blockade. If the magnitude of sympathetically-controlled tachycardia represents an important mechanism for blood pressure regulation during orthostasis, then blockade of SNA-mediated chronotropic responses should elicit syncopal symptoms at lower orthostatic stress in both high and low tolerant subjects. To test this hypothesis, heart rate was measured and compared before and after propranolol administration in individuals with high and low LBNP tolerance (Convertino and Sather, 2000a). Unlike cholinergic blockade, cardiac sympathetic blockade was associated with a lower LBNP tolerance (Figure 6, left panel) and dramatic reduction in the reserve for eliciting an adequate elevated heart rate (Figure 6, right panel) in both high and low tolerant groups compared to the placebo LBNP condition. The significant reduction in LBNP tolerance is particularly interesting in light of increased total systemic peripheral resistance resulting from inhibition by propranolol of a vasodilator effect on vascular smooth muscle beta-adrenergic receptors (Convertino and Sather, 2000a), although the reserve for vasoconstriction was unaltered (Figure 6, middle panel). As such, the results of this experiment demonstrated that the increase in heart rate elicited by cardiac SNA rather than peripheral vasoconstriction is critical to the sustainability of orthostatic competence in healthy individuals.

## Integration of vagal and sympathetic control of heart rate during orthostasis

Based on the evidence presented in this review, arterial vasoconstriction cannot by itself distinguish healthy individuals with high from those with low tolerance to orthostasis, or the reduction in tolerance induced by beta-adrenergic blockade. As such, a compelling argument can be made that the influence of elevated heart rate represents a prominent mechanism for maintaining adequate systemic perfusion pressure and consequently dictating orthostatic tolerance. This notion is most evident by the relationship between relative reductions in LBNP tolerance in high (−49%) and low (−39%) tolerant groups and corresponding tachycardic responses (−59 and -31%, respectively) during blockade of cardiac sympathetic influence.

**Figure 7:**
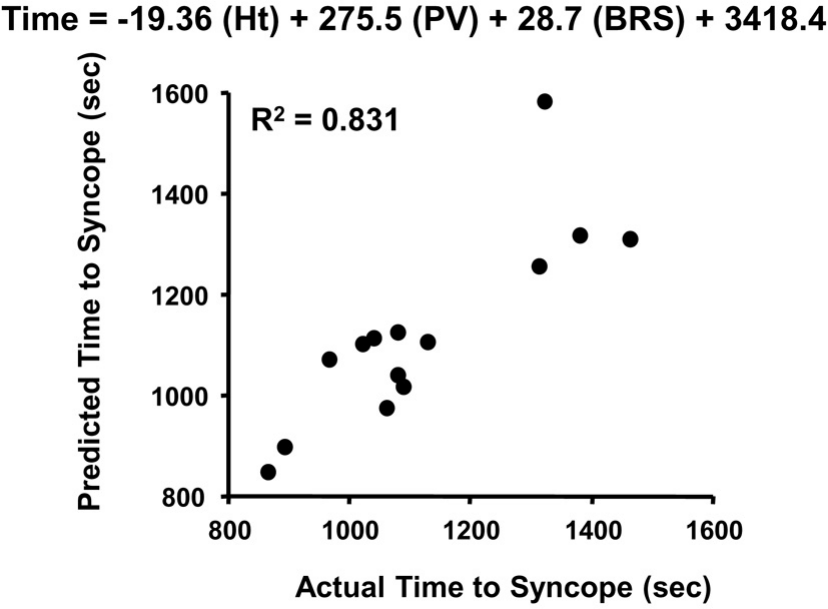
**Actual vs. predicted times to syncope based on the least squares model of height (Ht), plasma volume (PV), and cardiac baroreflex sensitivity (BRS)**. Figured modified from Ludwig and Convertino (1994).

The absence of change in LBNP tolerance with cardiac cholinergic blockade is perplexing given the overwhelming evidence of a consistent association of vagally-mediated cardiac baroreflex sensitivity and orthostatic hypotension. With this apparent discrepancy in experimental results, the interpretation of function of a reflex heart rate response mediated through parasympathetic control should be reconsidered. The initial response of multi-synaptic sympathetically-mediated reflexes (e.g., heart rate, vasoconstriction) requires 2–3 s to occur (Eckberg and Sleight, 1992), and 5–10 s for a maximum response (Rowell, 1993; p. 42). This delayed sympathetic response has been best demonstrated in our laboratory using a squat-stand test that results in pronounced transient reductions in total peripheral vascular resistance (reactive hyperemia) and blood pressure during the initial 10 s of orthostasis (Convertino et al., 1998; Rickards et al., 2007). In contrast, there is significant tachycardia within the initial 10 s of standing from a 4-min squat position (Convertino et al., 1998) that can only be explained by a rapid stimulation of the vagally-mediated carotid cardiac baroreflex which requires only milliseconds for a heart rate response (Eckberg and Sleight, 1992; Eckberg et al., 1992). It is likely that the maintenance of cardiac output and adequate tissue perfusion pressure during the initial moments of a rapid onset of gravitational challenge (e.g., lying or sitting to standing) is dependent on an important contribution of rapid cardiac parasympathetic withdrawal. This is supported by our observation that the carotid cardiac baroreflex was the only cardiovascular reflex represented in a multivariate regression model for predicting the onset of presycope in a rapid LBNP profile (Figure 7). The unique contribution of the carotid cardiac baroreflex to blood pressure regulation during orthostasis was further validated in our later experiments in which tolerance to rapid onset rates of LBNP and centrifugation was associated with cardiac baroreflex sensitivity in human subjects (Ludwig et al., 1998). Taken together, the evidence suggests that an integrated control of heart rate requires a rapid (parasympathetic) response to provide adequate tachycardia during the initial onset of orthostasis followed by an extended (sympathetic) maintenance of elevated heart rate. This hypothesis can be effectively tested with experiments designed to assess the heart rate and blood pressure responses with and without atropine and propranolol during the initial 10 s of an orthostatic challenge.

## Intergrated hemodynamic responses and orthostatic tolerance

The regulation of arterial blood pressure during orthostasis in healthy individuals represents a complex physiological system of integrated and redundant mechanisms. In this review of the role of heart rate response in the etiology of orthostatic hypotension in healthy individuals, there is no intention to suggest that mechanisms that influence the control of cardiac output and systemic peripheral resistance do not contribute importantly to the maintenance of orthostatic blood pressure. On the contrary, there are compelling data from our laboratory indicating that circulating vascular volume, preload, and vasoconstriction are important hemodynamic characteristics that can dictate the disposition for presyncope (Sather et al., 1986; Convertino, 1993; Engelke et al., 1994; Ludwig and Convertino, 1994; Convertino and Sather, 2000b). One contention is that an elevated heart rate will be accompanied by a reduced stroke volume since both are not necessarily independent of each other (Hainsworth, 2000). The caveat to this position, particularly under cardiac blockade, is that cardiac inflow remains unchanged (Weissler et al., 1957). Contrary to this notion, we have demonstrated that high tolerant subjects display higher cardiac inflow (filling) as evidenced by >30% greater end-diastolic volume (Sather et al., 1986). Consequently, average stroke volume in high tolerant individuals is similar to or slightly higher compared with low tolerant subjects (Sather et al., 1986; Rickards et al., 2011; Convertino et al., 2013). This cardiac relationship allows the high tolerant subjects to sustain orthostatic stability for a longer time at a lower stroke volume while maintaining similar maximal cardiac output (Sather et al., 1986). The finding that sympathetic cardiac blockade fails to create the same LBNP tolerance in both high and low tolerant individuals despite attenuated tachycardic response indicates that the onset of orthostatic hypotension and presyncope are influenced by other mechanisms (e.g., control of stroke volume and peripheral resistance). However, the evidence accumulated from a series of experiments conducted in our laboratory demonstrates the importance of cardiac chronotropic mechanisms in the development of orthostatic hypotension when the impacts of stroke volume and systemic vascular resistance are similar across experimental conditions.

## Summary

In this review, evidence gathered from numerous experiments conducted in our laboratory provided compelling evidence that reaffirms an important contribution of elevated heart rate in blood pressure regulation during orthostasis in healthy individuals. The notion that sympathetic- and parasympathetic-mediated tachycardia represents a significant underlying mechanism of orthostatic tolerance was consistent with two distinct observations: (a) despite similar peripheral vascular resistance, individuals with high LBNP tolerance have greater heart rate response than low-tolerant subjects; and (b) LBNP tolerance is significantly reduced when the heart rate response to LBNP is attenuated. Although not conclusive, evidence is presented to support the notion that cardiac parasympathetic withdrawal contributes to protection against reduced cardiac output and subsequent development of hypotension during the initial seconds of transition to an orthostatic challenge, while the primary mechanism by which tachycardia defends stability for extended durations of orthostasis is mediated predominantly through sympathetic adrenergic control.

### Conflict of interest statement

All experimental procedures were conducted under protocols reviewed and approved by the respective NASA, U.S. Air Force and U.S. Army Institutional Review Boards, and conducted in accordance with the approved protocols. The opinions or assertions contained herein are the private views of the author and are not to be construed as official or as reflecting the views of the Department of the Army or the Department of Defense. The author declares that the research was conducted in the absence of any commercial or financial relationships that could be construed as a potential conflict of interest.
